# Time Varying Encoding of Grasping Type and Force in the Primate Motor Cortex

**DOI:** 10.1523/ENEURO.0010-25.2025

**Published:** 2025-04-25

**Authors:** Adriana Moreno, Victor de Lafuente, Hugo Merchant

**Affiliations:** Instituto de Neurobiología, UNAM, Campus Juriquilla, Querétaro 76230, México

**Keywords:** grasping, motor cortex, movement preparation

## Abstract

The primary motor cortex (M1) is strongly engaged by movement planning and execution. However, the role of M1 activity in voluntary grasping is still not completely understood. Here we analyze recordings of M1 neurons during the execution of a delayed reach-to-grasp task, where monkeys had to actively grasp an object with either a side or a precision grip, and then pull it with a low or high amount of force. Single cell and neural populations analyses showed that grip type was robustly and specifically encoded by a large population of neurons, while force level was weakly and transiently encoded within mixed-selective neurons that also encoded grip type. Notably, the grip type was stably decoded from motor cortical populations during the preparation and execution epochs of the task. Our results are consistent with the idea that planning and performing specific grasping movements are high-level skills that strongly engage M1 neurons, while the execution of pulling force might be prominently encoded at lower stages of the motor system.

## Significance Statement

Grasping behavior requires precise motor coordination exerted by multiple brain areas, including the primary motor cortex (M1), but the exact role of M1 in grasping preparation and execution remains elusive. Here, we analyzed the neural activity of M1 while two monkeys performed a delayed reach-to-grasp task. We found that two parameters of grasping, grip type and pulling-force level, were encoded in the activity of single neurons and the neural population, although with important differences. While grip coding was stronger and more temporally stable, force encoding was weaker and short lived. Our results suggest that grip planning and execution is a high-level neural process that takes place independently of force control in M1.

## Introduction

Primates, including humans, often manipulate objects using their sophisticated prehensile hands. After determining an object's position in space, we must estimate object features like size, shape, texture, and weight. This information is then used by the brain to plan and execute precise movements to hold it appropriately. These movements comprise two main phases: a reaching phase in which the hand approaches the object and a grasping phase that occurs once contact is made. During the reaching phase, as the hand moves, the position of the fingers changes, adapting to features of the object we intend to grasp (preshaping). During the grasping phase, the position of the hand keeps adapting as the brain receives feedback from tactile and proprioceptive inputs (Jeannerod et al., 1995). Human and monkey hands are capable of different types of grips, including a highly controlled precision grip, in which an object is held between the palmar aspects of the fingers and the opposing thumb (Almécija and Sherwood, 2016). Besides controlling hand position and its derivatives, it is also crucial to regulate the amount of grasping force. If we apply too little force, the object may slip out of our hands, whereas if we apply too much, it may break or become damaged. Throughout the years, efforts have been made to determine how the brain gives rise to this highly precise and controlled behavior.

Due to M1's spinal projections, which include direct monosynaptic connections to spinal motoneurons, earlier studies aimed to find correlations between M1 neural activity and muscle activation (Strick et al., 2021). Surprisingly, the responses of M1 neurons showed to be highly diverse. While the firing rates of some neurons do correlate with muscle activation during single-joint movements and isometric force control (Cheney and Fetz, 1980; Evarts, 1968; Maier et al., 1993; Wannier et al., 1991), others seem to carry information about parametric aspects of reaching kinematics like direction, speed, trajectory, and dynamic force changes (Georgopoulos et al., 1986, 1992, 2007; Hatsopoulos et al., 2007; Inoue et al., 2018; Merchant et al., 2004; Naselaris et al., 2006a,b). In addition, recent studies have revealed that M1 neural populations can be described as a dynamical system in which internally generated temporal patterns generate motor commands that are sent to the spinal motoneurons and eventually, effector muscles (Churchland et al., 2012; Russo et al., 2018). In addition, it has been shown that M1 activity correlates with movement preparation, with population responses that converge to a particular state space when the system has encoded all the properties of the upcoming reaching movement (Afshar et al., 2011; Churchland et al., 2006; Riehle and Requin, 1993).

The motor cortex is also part of a cortical network that controls the visuomotor transformations for goal related grasping movements. This network includes, in hierarchical order, the anterior intraparietal area, areas 7a and 7ab of the posterior parietal cortex, ventral premotor cortex (F5p), and M1. Neurons in this network encode the visual features of the objects to be grasped, as well as the movement parameters associated with the type of grip needed for grasping (Brochier and Umiltà, 2007). Indeed, M1 neurons are not only tuned to different types of grips (Lemon et al., 1986; Mason et al., 2002) but also to the force exerted to grasp an object (Intveld et al., 2018). Nevertheless, grasping type recruits motor cortical activity to a larger degree as compared with grasping force, with a mostly independent control of the two parameters (Hendrix et al., 2009; Rastogi et al., 2021). In addition, M1 is also involved in the preparatory phase of grasping commands, where the population dynamics adopt different state trajectories during the preparation for different types of grasping movements (Meirhaeghe et al., 2023).

Here, we performed encoding and decoding analyses on the activity of simultaneously recorded M1 neurons from two monkeys performing a delayed reach-to-grasp task (Brochier et al., 2018). As shown before, we corroborate that at the single-unit and population levels, grip type had a profound effect on motor cortical activity, while force level explained less variance of the neural responses. We found that this was achieved through a combination of more neurons representing grip, as well as enhanced grip coding at the single-unit level. Notably, our analyses show that several neurons were modulated only by grip type, while force was encoded by neurons selective for both grip and force. Furthermore, the neural signals linked to grip type emerged right after grip instruction and were maintained after movement onset, forming a long-lasting and stable preparatory signal for precision or side grip that extended through movement execution. Importantly, even while pulling the object, when force level and grip type are simultaneously executed, force level transiently engaged fewer neurons and accounted for a small proportion of activity variance.

## Materials and Methods

### Behavioral task

**Figure 1.**
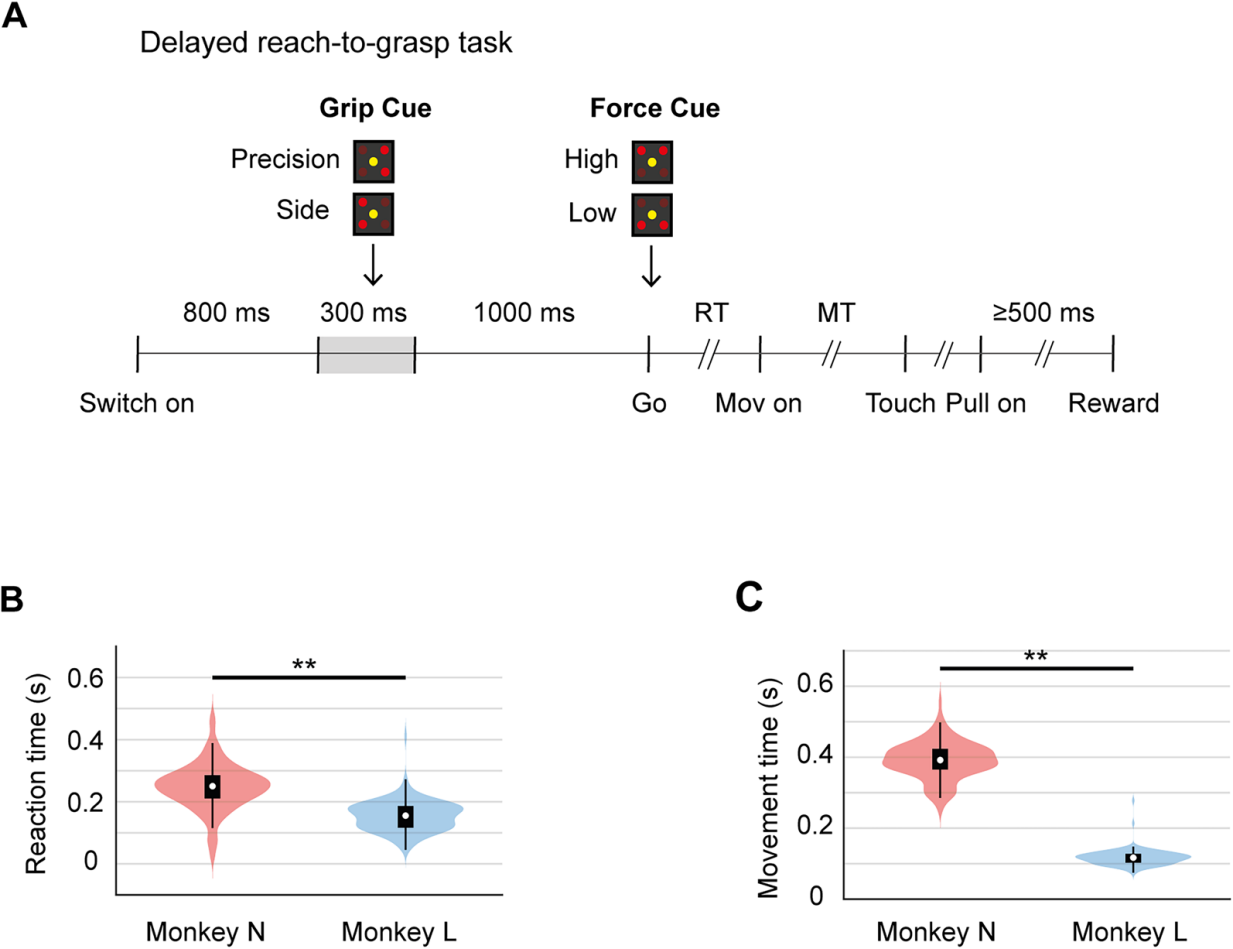
Delayed reach-to-grasp task. ***A***, Monkeys are instructed to grasp and pull an object using a specific hand grip and force level. Each trial starts with the monkey placing its working hand over a table switch (Switch on). After 800 ms, a visual cue indicates the type of grip (precision grip or side grip). After a 1,000 ms delay, another visual cue (Go) instructs the monkey to start the reach movement, as well as the amount of force (low or high) needed to pull the object. Reaction time (RT) was measured as the time between the Go cue and the movement onset (Movement on). Movement time (MT) was defined as the time between the movement onset and the object touch (Touch). Once grasped, the monkey must pull the object (Pull On) for at least 500 ms with the instructed force level. If the monkey performs correctly, it is rewarded with a drop of apple sauce (Reward). ***B***, Reaction times during correct trials for Monkey N and Monkey L, respectively. ***C***, Movement times for both monkeys, displayed as in ***B***.

We analyzed the online database generously made public by Brochier et al. (2018), who designed and performed all the experiments shown here. The task and recording methods are explained in detail previously (Brochier et al., 2018). Briefly, two macaque monkeys (*Macaca mulatta*), N (male) and L (female), were trained in a delayed reach-to-grasp task, in which they were visually instructed to grasp an object using either a side grip or a precision grip. After grasping, the monkeys had to pull the object for at least 500 ms with either a low or a high force level. From now on, we will refer to the amount of force required to pull the object as “force.” Monkeys were rewarded with a drop of apple sauce at the end of the trial if they followed the grip and force instructions correctly. Figure 1 shows the sequence of events that defined each trial of the reach-to-grasp task. Note that grip type and force level cues were temporally separated. The monkeys were first instructed about grip type, and after a 1 s delay, they were given an additional visual cue to indicate force magnitude. The force level cue also served as the go-cue to initiate the hand movement. Thus, monkeys had an established time window of 1 s (delay period) for grip preparation, while no explicit epoch was designated for force preparation, forcing the animals to prepare force level “on the go,” as the arm was moving. This also meant that, while grip preparation could be temporally isolated, grip execution as well as force planning and execution overlapped during the movement period of the task. Reaction time (RT) was defined as the time elapsed between the go-cue and the start of the reaching movement. Movement time (MT) was calculated as the time from movement onset until the object was touched.

### Neural recordings

Neural recordings were collected and preprocessed by Brochier et al. (2018). Briefly, a 10-by-10 Utah electrode array (Blackrock Microsystems) was chronically implanted in the primary motor (M1) and premotor cortex (dorsally in Monkey L and more ventrally in Monkey N) on the right hemisphere of each monkey, contralateral to the working hand. Neural signals from a total of 96 electrodes were amplified, high-pass filtered (0.3 Hz–7.5 kHz), and digitized with a sampling rate of 30 kHz. Raw signals were online high-pass filtered at 250 Hz and spike waveforms were detected by threshold crossing (manually set). To isolate single-unit activity, both online and offline sorting were performed. Manual online sorting was performed first for inspection, using Central Suite (Blackrock Microsystems). For all the analyses shown here, we considered only isolated units resulting from the offline sorting performed by the data providers (Brochier et al., 2018). Offline sorting was performed using Plexon Offline Sorter (Plexon). Cross-threshold unsorted waveforms were split into clusters in a two- or three-dimensional principal component space. *K*-means and Valley Seeking algorithms were used to sort waveforms coming from different units. Further inspection using interspike (ISI) distributions and autocorrelation and cross-correlation plots was performed to differentiate single-unit from multiunit activity. Since we focused only on motor cortical activity, we considered neural signals coming only from electrodes located posterior to the putative border between M1 and the premotor cortex (61 sites for Monkey N and 68 sites for Monkey L). In total, we analyzed neural data coming from 110 isolated units in the case of Monkey N and 97 units for Monkey L. We analyzed a total of two sessions (one session per animal) consisting of 137 trials for Monkey N and 135 trials for Monkey L. We considered only correct trials for all the analyses shown.

### Single-unit analysis

To study activity at single-unit level, we calculated the firing rate of each neuron as a function of time by using a causal exponential filter with a decay rate of 100 ms displaced every 20 ms. For each neuron, firing rates were averaged across trials and aligned to the onset of the reaching movement.

To characterize firing rate modulations of individual neurons in response to grip and force conditions, we calculated the area under the curve of the receiver operating characteristic (auROC) as a function of time. The auROC estimates the overlap between two distributions. For each neuron, the distributions compared are the firing rates of one condition versus its counterpart condition (side grip trials vs precision grip trials; and high force trials vs low force trials). An auROC value of 0.5 indicates that distributions completely overlapped and thus, firing rates are not useful to differentiate the two conditions. We calculated each neuron's auROC as a function of time and then performed a nonparametric permutation test to identify time points at which auROC was different from 0.5 (*p* < 0.001). In addition, if a neuron had a significant auROC for at least two consecutive time bins (permutation test for multiple comparisons, *p* < 0.001), then it was considered to significantly encode the tested parameter (de Lafuente and Romo, 2006; Merchant et al., 1997).

To determine whether single cell grip and force encoding were independent from each other, we first measured whether their modulation directionality was dependent on each other. For this, we performed a *X*^2^ test that compared the expected versus the observed marginal distributions of the grip and force auROC values for all cells and for each time bin (Fig. 4, middle panels) throughout the entire time series of the task. The expected marginal is the product of the observed probability of grip type (Fig. 4*A–D*, right scatter yellow distribution on the right) by the observed probability of pulling force (Fig. 4*A–D*, right scatter purple top distribution), while the observed marginal corresponds to the probability of observing both parameters in the actual data [computing the 2D histograms of the combined distributions (yellow and purple)]. In addition, we computed the 2D normal distribution on this data, obtaining ellipses whose angle can reveal dependency between parameters, where an angle close to 90° would indicate independence.

Next, we performed a correlation analysis to determine whether the modulation strengths of both parameters covaried. For this, we first estimated the ΔauROC, which is the absolute value of the difference between each neuron's auROC and 0.5. We then performed a nonparametric correlation test, in which we shuffled ΔauROC data across both time and neurons (1,000 times, without replacement). We obtained the Pearson’s correlation coefficient of each shuffle (between shuffled “force” ΔauROCs and shuffled “grip” ΔauROC values at each time point). We considered these null distributions and then estimated the true Pearson’s correlation coefficient between grip ΔauROCs and force ΔauROCs as a function of time and considered it significant when *p* < 0.001 for at least two consecutive time bins (permutation test for multiple comparisons, *p* < 0.001).

In addition to these tests, we also performed a cross-temporal correlation analysis, in which we compared grip ΔauROCs at each time point versus lagged force ΔauROC values (ranging from −400 to 400 ms, relative to the tested grip bin). The obtained Pearson’s correlation coefficients were tested against a shuffled (null) distribution (1,000 repetitions, no replacement, *p* < 0.001), for each time bin and lag. Correlations were considered significant if at least two consecutive time bins were significantly correlated (permutation test for multiple comparisons, *p* < 0.001).

### Neural population analysis

To determine whether the neural population of M1 encodes grip type and force level, we performed a demixed principal component analysis (Kobak et al., 2016). Similarly to principal component analysis (PCA), dPCA is a dimensionality-reduction method that allows to extract the neural population components that explain most variance (Gámez et al., 2019). In dPCA these components are referred to as “demixed” because they explain variance attributed to specific task variables. In our case, the demixed principal components (dPCs) were associated with grip type, force level, and temporal events within the task (referred to as condition-independent activity). Additionally, to detect the time points at which task parameters were significantly encoded by the neural population at the single-trial level, three linear classifiers were constructed using the first dPCs (i.e., that explained most variance) associated to each variable of interest (grip, force, and interaction) to decode task conditions. For each classifier, a stratified Monte Carlo leave-group-out cross-validation was performed. Monte Carlo chance distribution was set to 100 and time periods when the actual classification accuracy exceeded shuffled decoding accuracies in at least 10 consecutive time bins were considered significant.

### Neural population decoding

A decoding analysis was implemented using a methodology developed by Crowe et al. (2014), and Merchant and Averbeck (2017). For each neuron, the discharge rate was computed in 100 ms time bins within −2,000 to 2,000 ms relative to movement onset, resulting in 51 time bins, across the 30 trials of the two grip and two force conditions. For each time bin, we built an SVM classifier with a 10-fold cross-validation. The procedure was repeated 50 times using different training (*n* = 25) and testing (*n* = 5) trials each time, and the prediction results were averaged between repetitions. Accuracy thus estimated classifier's ability to discriminate the experimental conditions and corresponds to the total correctly predicted trials divided by the total number trials for each tested condition (2 for grip type or 2 for pulling force). A 50% accuracy corresponds to a random classification performance for the two levels of each parameter.

We carried out a cross-temporal decoding analysis in which we trained and tested the SVMs using all possible combinations of 100 ms time bins to establish the temporal evolution of grip and force coding (Mohan et al., 2021; Pearl et al., 2024). This analysis results in a classification accuracy matrix where the values along the diagonal are calculated by performing training and testing on equivalent time bins (Figs. 6*B*, 7*B*). In contrast, different time bins are used for training and testing and calculating the off-diagonal accuracy values. The off-diagonal accuracy allows us to determine how stable in time is a neural population signal when compared with the on-diagonal values. In fact, we considered an off-diagonal accuracy bin-pair combination as static, namely, a bin combination that shows decoding generalization from the on-diagonal bin classification model, when the three following conditions were met. The off-diagonal accuracy was significantly higher than chance (cluster-based permutation test, *p* < 0.01) and was above the 99% of the bootstrapped null distribution (1,000 iterations with shuffled classification labels), and the two corresponding time bins on-diagonal showed a classification accuracy above chance level (permutation test, *p* < 0.01, Bonferroni’s corrected for the number of on-diagonal time bins). Next, we computed a binary matrix of the same size as the cross-temporal decoding matrix, where we assigned 1 to at least two consecutive time bins resulting as static and 0 for all remaining time bins. Then, we quantified the magnitude of cross-time decoding between and within the two main task epochs using the generalization index. The generalization index provides information about the proportion of SVM tested bins during the preparation and execution that were classified as static during the trained bins of either the preparation or execution epoch.

### Code accessibility

The dataset analyzed in this paper was collected, described, and made public by Brochier et al. (2018) and can be freely accessed using the following link: https://doi.gin.g-node.org/10.12751/g-node.f83565/. The custom MATLAB code used to perform the analyses shown here is also freely available (https://github.com/MerchantLabINB/GraspForce2025).

## Results

### Monkeys were able to use two grip types and two force levels to grasp and pull an object

In the delayed reach-to-grasp task, monkeys were instructed to grasp a cubic object using either a side or a precision grip. Once grasped, the monkeys had to pull and hold the object for at least 500 ms with a low or high amount of force. Figure 1*A* shows the main events of the task. Each trial started with the monkey placing its left hand over a table switch. After 800 ms, a visual cue was displayed for 300 ms, instructing the monkey about grip type. After a 1,000 ms delay, an additional visual cue indicated the required force level to pull the object. This cue also served as a go cue for the monkey to start the reaching movement. Thus, this task was designed to determine the neural underpinnings of movement preparation and execution of multijoin complex hand movements, and the neural signals related with the execution of two types of grasping movements combined with two levels of pulling force.

The behavioral results show that Monkey L performed the task faster, with shorter and less variable reaction times (Fig. 1*B*; mean ± SD: 150 ms ± 48 ms) compared with Monkey N (mean ± SD: 251 ms ± 190 ms). Movement times were also shorter and less variable in Monkey L as compared with those in Monkey N (Fig. 1*C*; mean ± SD; Monkey L, 123 ms ± 80 ms; Monkey N, 343 ms ± 125 ms). Both parameters were significantly different between animals (reaction time, *z* = −11.51, *p* = 1.07 × 10^−30^; movement time, *z* = −14.01, *p* = 1.29 × 10^−44^, Mann–Whitney *U* test).

### Grip type has a larger influence in M1 neurons compared with force level

**Figure 2.**
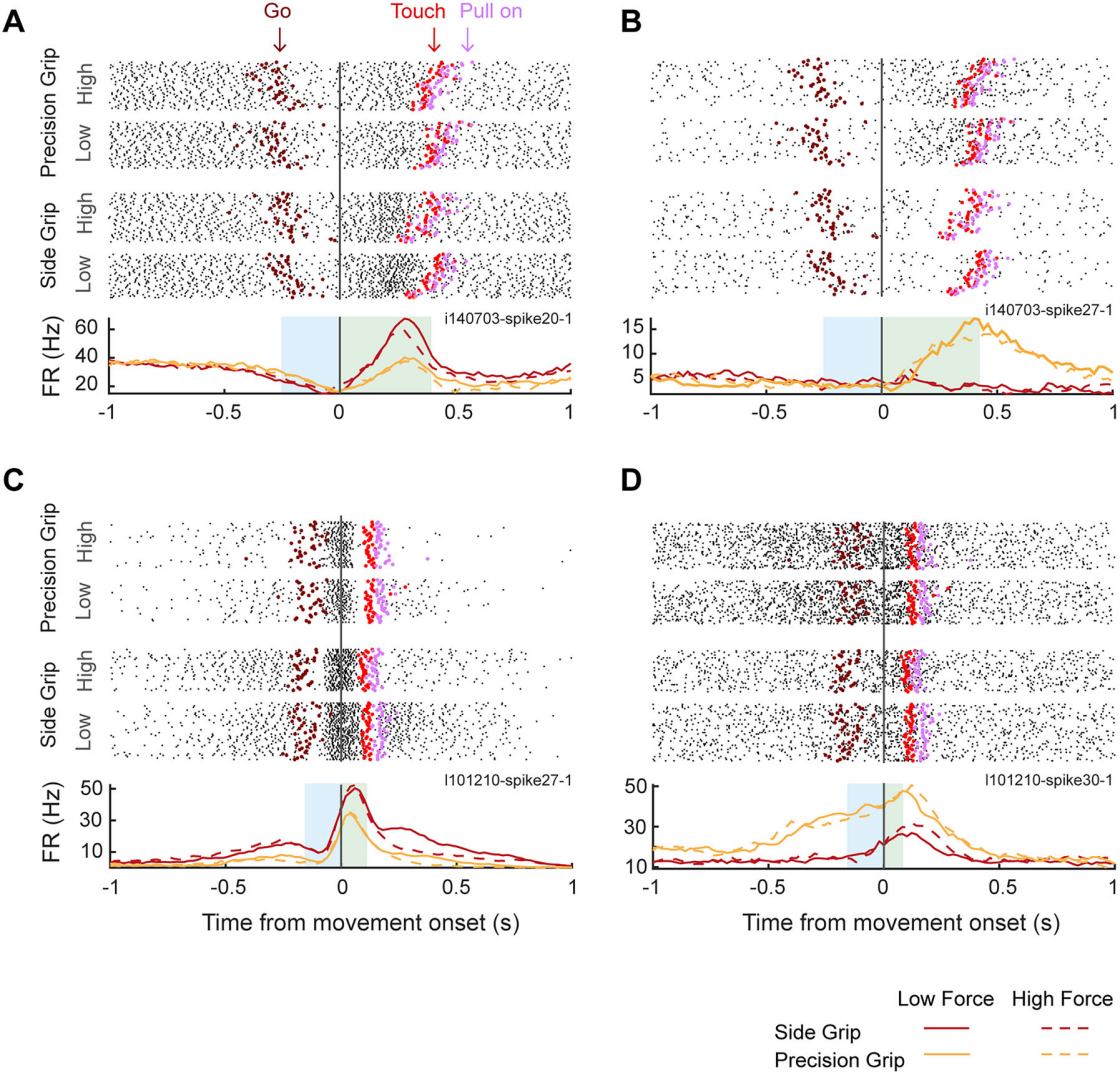
Four example neurons of M1 during the delayed reach-to-grasp task. ***A***, ***B***, Top, Raster plot of two example neurons from Monkey N. Trials are grouped according to the instructed grip type and force level, as indicated by the legends on the left (from bottom to top: side grip and low force, side grip and high force, precision grip and low force, precision grip and high force). Activity is aligned to movement onset (gray solid line). Colored markers indicate task events around movement onset: Go cue (dark red), object touch (red), pull onset (lilac). ***A***, ***B***, Bottom, Trial-averaged firing rate across task conditions. Line color indicates grip type (red for side grip and yellow for precision grip). Line style indicates force level (dashed line for high force and solid line for low force). Blue shading indicates the mean reaction time and green shading indicates mean movement time. ***C***, ***D***, Activity of two example neurons from Monkey L (same conventions as in ***A*** and ***B***).

We analyzed a total of 110 units for Monkey N and 97 units for Monkey L. Figure 2*A* shows the raster plot and firing rate of a neuron from Monkey N that increased its activity after movement onset, with a preference for side over precision grip trials. Note that, as the monkey's hand approached the object (object touch), activity started to become modulated also by force level. Figure 2*B* shows a neuron that fired strongly during precision grip trials. Figure 2*C* illustrates a neuron that fired more during the preparation and execution of a side grip and a low force level. Finally, Figure 2*D* depicts a neuron that is more active during preparation and execution of a precision grip and a high force level.

**Figure 3.**
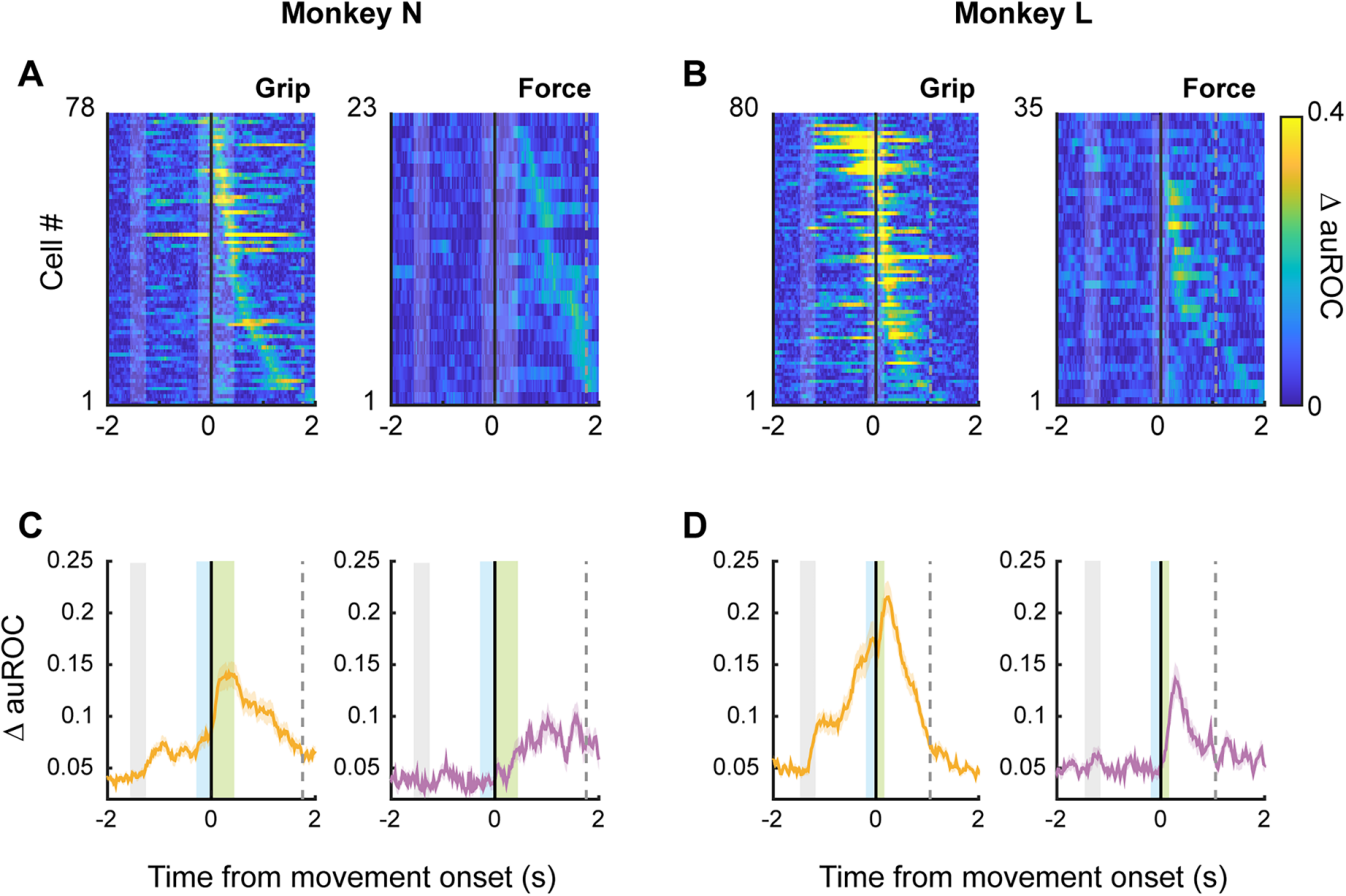
M1 single units are modulated by grip type and force level. ***A***, Heatmaps of grip (left) and force (right) coding in single units, using the area under the curve of the receiver operating characteristic (auROC) for Monkey N. Only neurons with significant tuning for each parameter are shown (auROC significantly away from 0.5; permutation test, *p* < 0.001). ***B***, Same as in ***A***, for Monkey L. ***C***, Average grip (left) and force (right) ΔauROC across neurons as a function of time for Monkey N. Gray shading indicates the duration of the grip cue. Blue and green shadings indicate mean reaction and movement times, respectively. Black solid line indicates movement onset. The gray dashed line indicates the mean object release time. ***D***, Same as in ***C***, for Monkey L.

To determine whether single-unit activity contained information about task parameters, we computed the auROC, comparing side versus precision grip trials (grip) and low versus high force trials (force; see Materials and Methods). Neural activity was aligned to movement onset. The heatmaps in Figure 3*A*,*B* show the absolute value of the difference between auROC values and 0.5 (ΔauROC), for neurons with significant firing rate modulations for either grip type (left panel) or force level (right panel; permutation test, *p* < 0.001). Neurons were sorted by the time of their maximum ΔauROC value. In Monkey N, the average ΔauROC peaked after movement onset for most neurons, while maximum force ΔauROC occurred later, after object touch. It is worth noting that grip- and force-encoding peaks span large temporal windows, and this can be interpreted as a dynamic activation chain in which neurons successively encode movement parameters, with especially brief selective periods for force level. This type of population dynamics has been reported in numerous cortical areas during different behavioral tasks (Crowe et al., 2014; Merchant et al., 2015; Mendoza et al., 2018, 2024; Gámez et al., 2019). In addition, the time profile of ΔauROC clearly indicates that individual neurons were less modulated by force as compared with grip (Fig. 3*C*,*D*). Note also that Monkey L reached higher ΔauROC values for both parameters and exhibited a stronger preparatory signal for grip type, compared with Monkey N.

**Figure 4.**
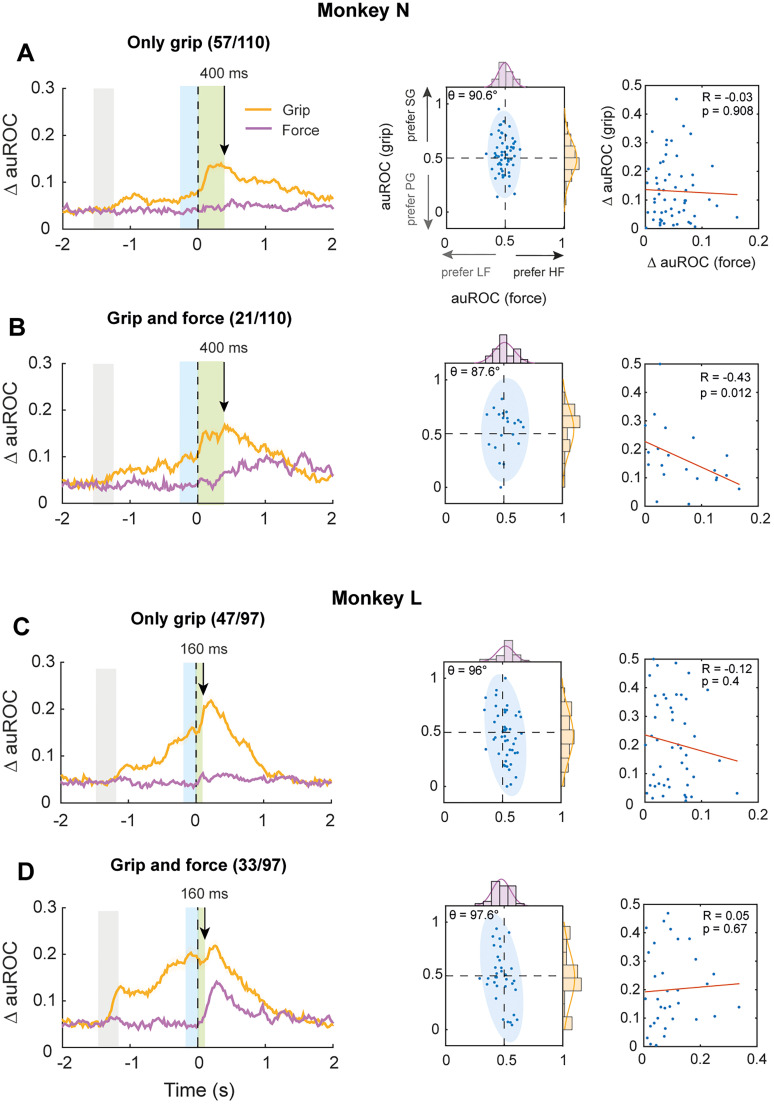
Grip and force neural modulations are not correlated. ***A***, Left, Average ΔauROC related to grip (yellow solid line) and force (purple solid line) as a function of time for neurons that showed significant tuning for grip type only (only grip), in Monkey N (permutation test, *p* < 0.001, *n* = 57 neurons). Middle, Grip as a function of force auROC values, for only-grip coding neurons, 400 ms after movement onset (object touch). Each blue dot represents a neuron. Blue ellipse represents the 95% confidence interval of the 2D distribution. *θ* is the angle of rotation of the ellipse. Purple and yellow histograms at top *x-* and right *y*-axes are the distributions of the auROC values for force and grip, respectively. Right, Grip as a function of force ΔauROCs for only-grip selective neurons, 400 ms after movement onset. Red solid line is the simple linear regression model fitted to the data. The correlation coefficient (*R*) and the *p* value (*p*) are indicated at the top right. ***B***, Same as in ***A***, for neurons that coded both grip and force significantly (permutation test, *p* < 0.001, *n* = 21 neurons) in Monkey N. ***C***, same as in ***A***, for Monkey L (160 ms after movement onset, only-grip neurons, *n* = 47. Permutation test, *p* < 0.001). ***D***, same as in ***B***, for Monkey L (160 ms after movement onset, grip-and-force neurons, *n* = 33. Permutation test, *p* < 0.001).

Next, we identified and grouped neurons according to the modulations in their firing rates by both parameters. In Monkey N, we found 57 neurons that were significantly modulated by grip type, while 21 were tuned to both grip and force. We found similar proportions in Monkey L, with 47 units being modulated only by grip, while 33 were mixed-selective for both parameters. In both animals, only a small number of neurons (*n* = 2, for each monkey) were significantly modulated by force only, indicating that most of the force encoding in M1 occurs through neurons that are also modulated by grip. Figure 4*A* (right) shows the average ΔauROC for grip and force as a function of time, across neurons that were modulated only by grip type, in Monkey N (left). The middle panel shows each neuron's grip auROC values as a function of force auROCs, 400 ms after movement onset for the same subpopulation. Notice how the rotation angle of the 95% confidence interval (blue ellipse) is close to 90°. This indicates that, while the firing of most of these neurons is modulated by grip type (auROC values uniformly distributed along the *y*-axis), the directionality of these modulations does not covary with force's (i.e., neurons that have preference for one grip do not necessarily have preference toward a specific force level). To formally test this, we performed an independence test, comparing the two marginal distributions of grip and force auROCs throughout the entire time series (see Materials and Methods). We did not find a significant dependence between the directionality of grip and force encoding at any time bin (permuted *X*^2^ test, *p* < 0.001). The same analysis was performed using units that encoded both grip and force and we found similar results (Fig. 4*B*). The absence of dependence between grip and force encoding was also observed in Monkey L (Fig. 4, middle panels *C* and *D*).

It is possible that, even when the directionality of firing rate modulations is independent between parameters, a correlation between the strength of these modulations exists. Therefore, we performed a correlation analysis between grip and force modulations, now comparing the absolute changes in auROC (ΔauROC) instead of their raw values. We did not find significant correlations between the strength of grip and force-related modulations at any time point in any of the neural subpopulations analyzed (permutation test, *p* < 0.001). Figure 4 (right panels) shows grip ΔauROC as a function of force ΔauROC values for each neuron during a time point close to the mean time when each monkey touched the object (400 ms for Monkey N and 160 ms for Monkey L). Lastly, we performed the same correlation analysis “cross-temporally,” comparing grip versus lagged force ΔauROC values (ranging from −400 to 400 ms). We found sparse and brief correlations (for three bins or less) in the mixed-selective subpopulations of both monkeys (data not shown).

These findings suggest that grip and force might be encoded as separate entities in motor cortical cells, even when individual units are modulated by both parameters. However, this effect may be partially affected by temporal differences between both signals, given that force encoding emerges later in the task.

### Population encoding of grip type and force level

**Figure 5.**
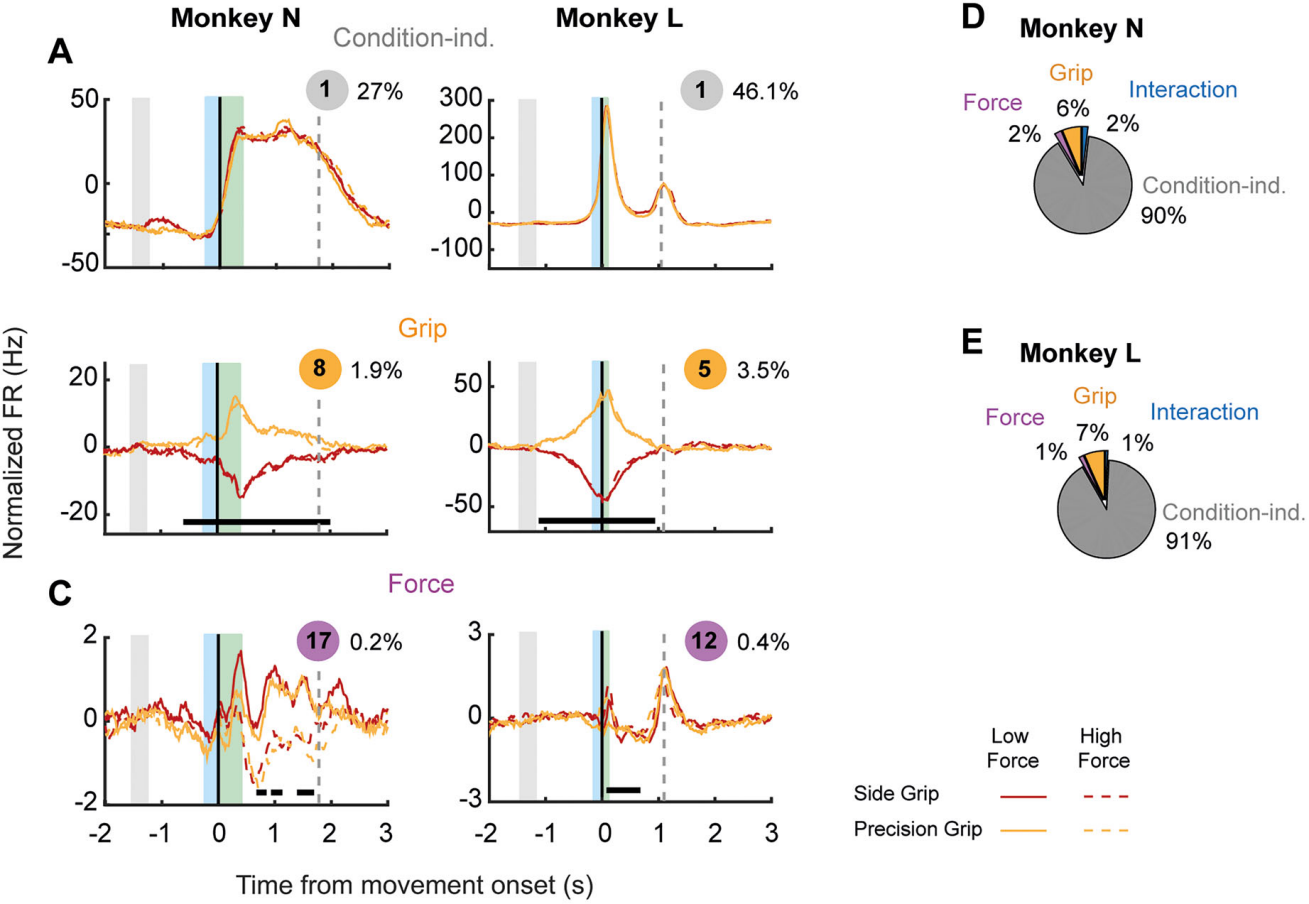
Population-level coding of grip type and force level in M1 using dPCA. Firing rates were projected onto the demixed principal component (dPC) that explains the most variance associated with each task parameter (condition-independent, grip and force). ***A***, First condition-independent component (component #1) for Monkey N (left) and Monkey L (right). ***B***, First significant grip component for Monkey N (component #8, left) and Monkey L (component #5, right). ***C***, First significant component associated with force for Monkey N (component #17, left) and Monkey L (component #12, right). Activity was aligned to movement onset (vertical black solid line). Gray shading indicates the duration of the grip cue. Blue and green shadings indicate mean reaction and movement times, respectively. Vertical dashed line corresponds to the average time of object release. Component number and percentage of explained variance are shown at the top right corner of each panel. Black solid line at the bottom indicates time points at which the task parameter could be decoded from the neural activity at single-trial level using its associated dPC as a classifier. ***D***, Percentages of total explained variance by each task parameter for Monkey N. ***E***, Same as in ***D***, for Monkey L. Correlations between main dPCs (condition-independent and force-related) and pulling-force signals were measured and are shown in Extended Data Figure 5-1.

We performed a population analysis using demixed principal components (dPCs) which estimated the percentage of variance associated with (1) condition-independent modulations (related to the temporal structure of the task), (2) grip type, and (3) force level. Figure 5*A* shows the normalized firing rates of all M1 neurons projected onto dPC #1 for Monkeys N (left) and L (right). We found that the largest proportion of population variance is explained by condition-independent activity that is related to the temporal structure of the task, i.e., to the moment at which the monkeys initiate and release the grasp on the object.

10.1523/ENEURO.0010-25.2025.f5-1 Figure 5-1 **Correlation between the monkeys’ pulling force and condition-independent and force-related dPCs from M1 neurons.** A: Normalized average pulling force signal, as a function of time for monkey N (left) and monkey L (right). B: Condition-independent (time) dPC that explained the most neural variance in monkey N and L, respectively. C: Force dPC that explained the most neural variance for monkey N (dPC #17) and monkey L (dPC #12). D (left): Absolute value of the maximum cross-correlation coefficient (R^2^) between each trial force signal and its related dPC (force and time), for low force (blue box) and high force (red box) trials, for monkey N. Black lines with asterisks indicate significant differences between marginalizations (2-way ANOVA, F(1,279) = 481.68, p = 0). D (right): Proportion of trials with a significant value (outside of the 99%-confidence bounds) of R^2^ when comparing the force signal between grip and force-related dPCs for monkey N. E: same as in D for monkey L (2-way ANOVA, F(1,327) = 233.35, p = 0). Download Figure 5-1, TIF file.

It is interesting to note that dPC #1 in Monkey N showed a less pronounced slope in the increase of activity associated with movement onset, and this might be related to the slower reaction and movement times of the animal compared with Monkey L. This increase was maintained during the entire movement period, with activity decreasing after object release. Conversely, neural temporal dynamics on Monkey L were overall faster, with a more pronounced slope during the perimovement period and a sudden drop after object touch. This was followed by a small increase in activity in response to object release.

The dynamics of the dPCs related to grip type are shown in Figure 5*B*. It must be noted that the amount of variance associated with grip type in both animals is an order of magnitude smaller compared with that related to the temporal structure of the task. In the case of Monkey N, dPC #8 explained 1.9% of total variance, but this was enough to decode hand grip at the single-trial level, before movement onset. For Monkey L, dPC #5 explained 3.5% of total variance and allowed to predict the monkey's hand grip hundreds of milliseconds before the animal received the go cue. Grip encoding was maintained until the monkey released the object and was not held any further, as opposed to what we observed in monkey N.

The dynamics of the activity related to force level are shown in Figure 5*C*. In Monkey N, dPC #17 explained 0.2% of total variance, and it allowed to decode force level later in the trials and for shorter time windows, compared with grip type. For Monkey L, dPC #12 allowed to predict force trials more consistently, from movement onset until hundreds of milliseconds before object release. Figure 5, *D* and *E*, shows the total explained variance for each task variable in Monkey N and Monkey L, respectively. In both cases, condition-independent components explained ∼90% of total neural variance. This was followed by grip that explained 6 and 7% of total variance in Monkeys N and L, respectively. Force explained only 2% of variance in the case of Monkey N and 1% for Monkey L. The interaction of grip and force explained <2% of total variance, and interestingly, none of its associated dPCs allowed above-chance decoding of task condition at the single-trial level; hence, they were not considered to be significant.

### Relation between behavior and neural decoding

To assess whether the neural population encoding captured by the main dPCs correlated with the actual pulling force, we measured the correlation between the exerted pulling force as a function of time and condition-independent and force-related dPCs from M1 neurons. First, it is evident that both monkeys performed the task properly, applying force only after the go signal and with no force changes during the delay period (Extended Data Fig. 5-1*A*). As expected, the exerted force level was higher during the high force trials. However, as evidenced by the dPCA analysis, the time profiles of force and time decoding varied between monkeys. While Monkey N exhibited more tonic neural modulations that resembled the sustained application of force, neural decoding of time and force was more phasic, differing from the tonic pulling-force signal. This comes in agreement with previous studies describing diverse time profiles of force-related neural responses that do not necessarily equal force application time (Cheney and Fetz, 1980; Wannier et al., 1991; Hendrix et al., 2009). Furthermore, the correlations between the applied force and the force dPCs were smaller than those between the applied force and the condition-independent (time) dPCs for both animals (Extended Data Fig. 5-1, panels *D* and *E*).

### Population activity allows decoding grip type and force level during single trials

**Figure 6.**
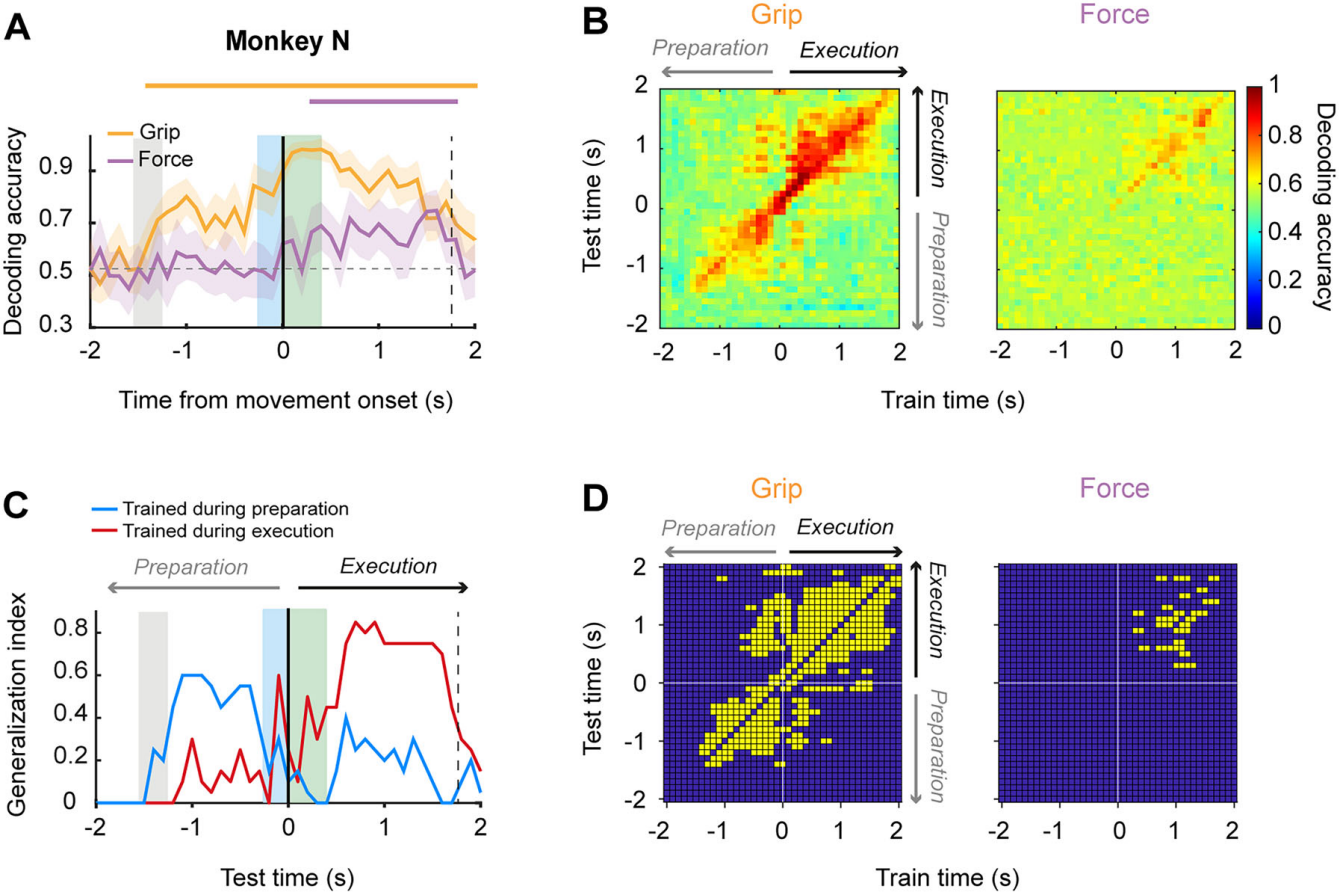
Temporal stability of grip and force encoding in M1 (Monkey N). ***A***, Decoding accuracy of grip type (yellow solid line) and force level (purple solid line) as a function of time using an SVM classifier. Solid lines at the top indicate the times at which accuracy was significantly above chance (*p* < 0.01). Neural activity was aligned to movement onset, indicated by the black vertical line. Gray shading indicates display of the grip cue. Blue and green shadings indicate mean reaction and movement times, respectively. The vertical dashed line corresponds to the average time of object release. ***B***, Cross-temporal decoding of grip (left) and force (right) during the perimovement period of the task. ***C***, Generalization index, estimated as the proportion of static bins resulting from the cross-temporal decoding of grip as a function of time when training and testing the classifier during preparatory and executive periods of the task. ***D***, Stable time points of the cross-temporal decoding of grip (left) and force (right). Yellow squares indicate significantly stable time bins.

**Figure 7.**
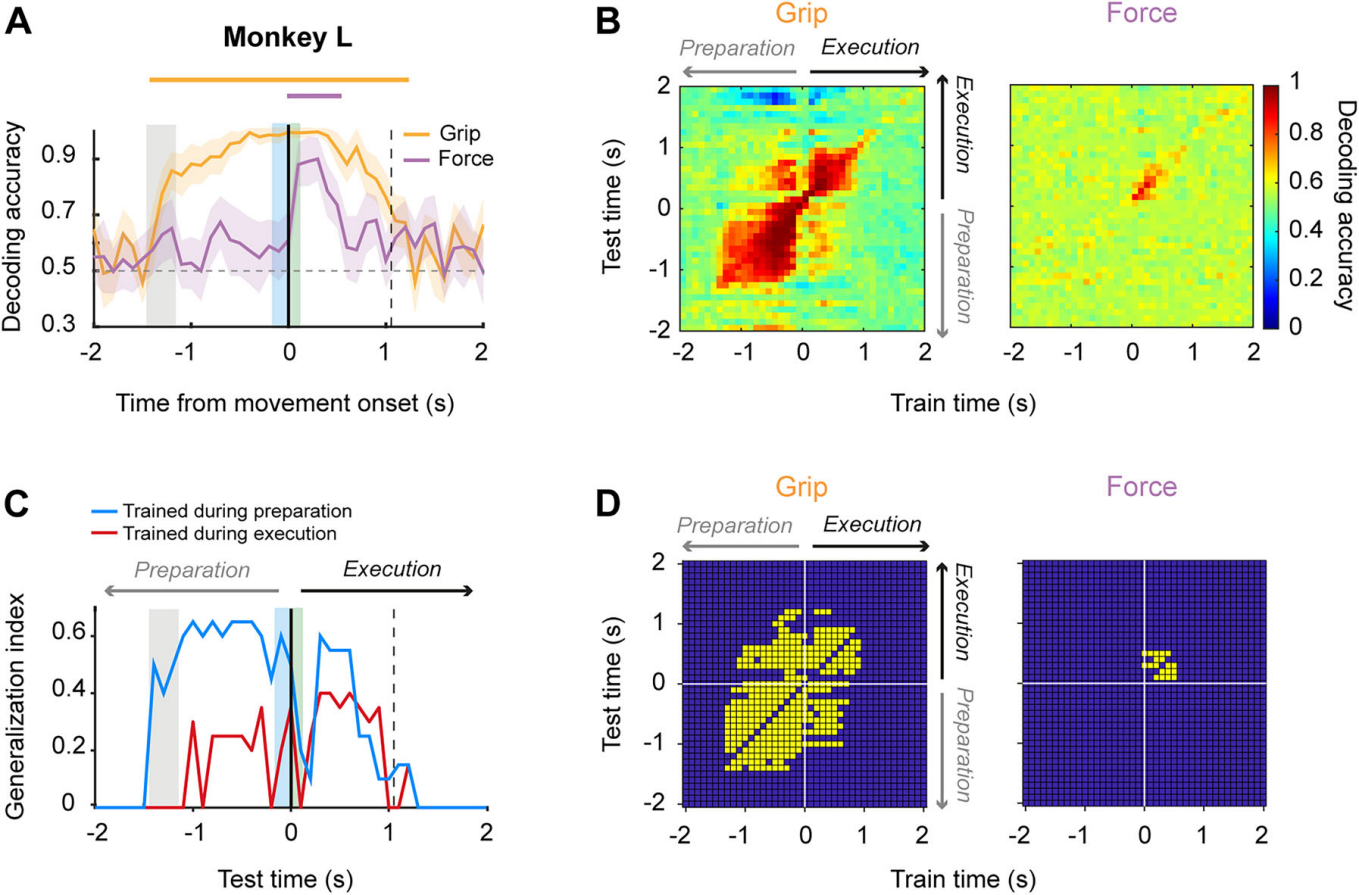
Temporal stability of grip and force encoding in M1 (Monkey L). ***A***, Decoding accuracy of grip type (yellow solid line) and force level (purple solid line) as a function of time using an SVM classifier. Solid lines at the top indicate the times at which accuracy was significantly above chance (*p* < 0.01). Neural activity was aligned to movement onset, indicated by the black vertical line. Gray shading indicates display of the grip cue. Blue and green shadings indicate mean reaction and movement times, respectively. The vertical dashed line corresponds to the average time of object release. ***B***, Cross-temporal decoding of grip (left) and force (right) during the perimovement period of the task. ***C***, Generalization index estimated as the proportion of static bins resulting from the cross-temporal decoding of grip as a function of time when training and testing the classifier during preparatory and executive periods of the task. ***D***, Stable time points of the cross-temporal decoding of grip (left) and force (right). Yellow squares indicate significantly stable time bins.

Given that the neurons were simultaneously recorded, we used SVM classifiers to test whether population activity allowed the decoding of grip type and force during single trials. Figures 6*A* and 7*A* show the decoding accuracy of the grip type and grip-force classifiers as a function of time in Monkeys N and L, respectively. For both animals, the above-chance decoding for grip type started almost immediately after the onset of the grip cue and continued during movement execution. In contrast, significant decoding of force level was present only during movement execution and its magnitude was smaller than for grip. Next, to assess how stable grip and force encoding were throughout time, we performed a cross-temporal decoding analysis. Specifically, we trained and tested both classifiers using all possible pairs of time bins during the peri-movement period of the task. This method produces a classification accuracy matrix where the off-diagonal values are a measure of how generalizable the neural encoding of grip type or force at a particular time bin is, when directly compared with the corresponding diagonal values, where the training (*x*-axis) and testing (*y*-axis) of the classifier are over the same time bin. Hence, the level of generalization across time bins tells us how consistently a set of neurons encode grip type or force across time during task performance. Figures 6*B* and 7*B* show the heatmaps of the cross-temporal decoding accuracies for grip and force classifiers using neural activity from Monkeys N and L, respectively. For Monkey L, grip decoding was more stable in time (static), with two generalization clusters, one during movement preparation, and another during grip execution. This is more evident in the binary matrix of Figure 7*D*, where the significant static off-decoding bins are depicted in yellow.

In contrast to grip type, force decoding was temporally less stable (dynamic), with only a few significant off-diagonal time bins, all of them close to the on-diagonal time bins, and that occurred only during the movement execution (Fig. 7*D*). In the case of Monkey N, grip type decoding was also more temporally stable than force (Fig. 6*B*). However, stable time bins were distributed more sparsely across preparation and execution periods, with more static bins clustered within the execution period of the task.

We developed a generalization index that corresponds to the proportion of SVM bins that were classified as static when cross-temporally decoding grip and force across the preparation and execution epochs of the task. Hence, these indexes summarize how the significant decoding during the preparation epoch generalizes within the preparation and for the execution or how the significant decoding during execution generalizes for the preparation and execution epochs. Figures 6*C* and 7*C* show the generalization index as a function of test time when the classifier was trained during preparation (blue line) and execution (red) for Monkeys N and L, respectively. It is evident that there is a generalization of grip type decoding from preparation to execution and vice versa, suggesting that the motor cortical populations possessed information about the type of grip across task epochs, although there is a clear bias for a larger number of static bins when training and testing the classifier in the same epoch. Interestingly, grip type generalization was more pronounced during preparation for Monkey N, whereas decoding during execution generalized more in Monkey L.

## Discussion

In this study we aimed to explore the role of primate's cortical area M1 during a reach-to-grasp behavioral task that required the precise regulation of two parameters: grip type and force level. Our findings support three conclusions. First, we found that at the single-unit and population levels, grip type had a larger effect on motor cortical activity, while the force level was weak and transiently encoded by single units and explained less neural variance. Second, grip type was encoded through movement preparation and execution, while force encoding was brief and emerged later during movement execution. Importantly, grip type was encoded by both grip-selective and mixed-selective cells, while force was encoded mostly by mixed-selective cells. Analyzing the marginal probabilities in the selectivity of cells we found no interaction between the two parameters, supporting the notion that grip type and pulling force are coded as separate entities in the motor cortex. The lack of significant interaction between grip type and pulling force in the demixed PCA corroborated this finding at the neural population level. Third, the neural modulations linked to grip type emerged right after grip instruction and were maintained after movement onset, forming a long lasting and stable signal for precision or side grip during the preparation and execution epochs of the task. Thus, the categorical grip signal was more static compared with force, which occurred later and was more phasic. Our findings support the notion that M1 is strongly engaged in encoding grip type, with neural populations distinguishing between the two grips across the preparation and execution epochs of the task. In contrast, force was encoded transiently during movement execution and was mostly independent of grip type.

### Grip type is encoded at both single-unit and population levels in M1

For both animals, most neurons were strongly modulated by grip. Neural coding of grip emerged during the delay period and peaked near movement onset. Since monkeys had to perform either a side or a precision grip, it is important to highlight the differences between them, especially in the motor coordination required for each. While a side grip demands the coordination of the thumb and the rest of the fingers as a group, a precision grip is a finer, and therefore a more controlled, movement that requires independent coordination of the fingers to grasp an object using only the thumb and the index finger (Edin et al., 1992; Jeannerod et al., 1995; Almécija and Sherwood, 2016). A previous study has shown that M1 neurons fire more during a precision grip compared with a power grip (Muir and Lemon, 1983). This neural modulation does not seem to represent muscle engagement exclusively, since there is not an absolute correlation between net force and the firing rate of individual neurons of the pyramidal tract in M1 (Muir and Lemon, 1983; Maier et al., 1993). A possibility is that action complexity and therefore, the level of motor control needed could modulate neural activity and send specific motor commands to the spinal cord related to different hand grips (Muir and Lemon, 1983). Whether neural activity in M1 represents muscles or movements has been a topic of debate for years. Evidence supports the notion that both can be equally represented in M1 neural activity (Kakei et al., 1999; Wang et al., 2022). Our results show that grip type was encoded at the neural population level and dPCA analysis of population activity demonstrated that, in both animals, grip encoding emerges during the delay period, while the animals prepare the movement. Similar to what we found at the single-unit level, significant decoding of grip was possible earlier in Monkey L, almost immediately after the grip cue. In addition, our findings at the neural population-level are consistent with what has been previously reported in the literature for M1 and other grasping-related areas like the anterior intraparietal area (AIP) and the anterior portion of the ventral premotor cortex (area F5), in which time is the most represented task parameter, followed by grip and lastly, force (Intveld et al., 2018; Rastogi et al., 2021).

### Force level is weakly encoded at both single-unit and population levels in M1

We found that force level elicited weaker modulations in the firing rate of individual neurons compared with grip type. Moreover, we found fewer force-coding neurons, compared with grip-coding neurons. Interestingly, most of these cells were comodulated by grip, which implicates that the coding of force in M1 occurs through mixed-selective neural subpopulations mostly (Rigotti et al., 2013; Kobak et al., 2016). At the population level, we found that force also explained less neural variance than grip, consistent with what we found in single units.

At first, we considered that a possible explanation for the differences between grip and force coding could reside on the time the animals had to prepare both. We thought of the possibility that having less time to prepare the appropriate force level could impact on the amount of explained variance within the neural population of M1. However, other studies using an extended version of this dataset found that, even when the monkeys are instructed first on the load force level (reverse condition), classification accuracy of force using local field potentials (LFPs) remains lower than grip (Milekovic et al., 2015). Moreover, a previous study using dPCA to characterize the population coding of grip type and grip force in grasping-related areas, including M1, also showed that grip force explained less neural variance than grip type, even when both grip and force were simultaneously planned within the same time window (Intveld et al., 2018). Therefore, our results agree with such previous findings.

Remarkably, the force-encoding subpopulations showed brief activation profiles with selectivity for the low-force and others for the high-force conditions, suggesting that force might not be encoded linearly in M1 neural activity. These M1 neural properties contrast with the steady pulling force exerted by the monkeys after movement onset, which was by task design, larger for the high force condition. Therefore, the transient and nonlinear encoding of force suggests that M1 might generate categorical signals that are read and transformed downstream to control the actual hand pulling force.

### Grip and force might be encoded as independent parameters in M1 at both single-unit and population level

Several studies have found that motor-related areas like the posterior parietal cortex (PPC), AIP, the dorsal and ventral premotor cortices (PMd and PMv, respectively), and M1 actively participate in grip and force control during grasping (Davare et al., 2006; Chib et al., 2009; Intveld et al., 2018). However, whether these aspects of grasping behavior are part of the same neural process, or if they are driven by different neural mechanisms, has been a topic of debate (Evarts, 1968; Tanji and Evarts, 1976; Cheney and Fetz, 1980; Georgopoulos et al., 1986; Taira et al., 1996; Churchland et al., 2012). While some studies have pointed to a common neural substrate for grip and force control in M1 (Maier et al., 1993; Hepp-Reymond et al., 1999), more recent findings suggest that these parameters might be represented independently of each other in motor and premotor areas in both single units and the neural population (Hendrix et al., 2009; Intveld et al., 2018). Accordingly, our findings demonstrate that the neurons that were selective for both grip type and pulling force covered the four possible encoding scenarios (side/precision grip, low/high force) with no correlation in both the modulation directionality and strength of the two parameters across motor cortical neurons. Furthermore, a lack of dependence between grip and force encoding could also be seen at the population level, with no significant interaction between grip and force encoding, as evidenced by the dPCA. Taken together, our results support the idea that the motor cortex may maintain the neural signals for grip and force as separate entities mostly. However, to fully corroborate this, further observations regarding this feature should be made in tasks in which grip type and force level planning times are similar.

### Grip encoding is more stable than force in M1

Lastly, we evaluated the temporal stability of grip and force encoding. We found that grip coding was stable in time (hence, static) during the preparation and execution epochs of the task. The cross-temporal decoding analysis suggests that similar populations of neurons maintained information about the grip type for hundreds of milliseconds in both epochs of the task. In addition, since the analysis of the applied force revealed that monkeys only exerted grasping force after the go cue, this analysis supports the notion that grasping-selective responses during the delay period are linked to movement preparation. The neural mechanisms behind the static coding scheme are still being studied. However, it has been proposed that neural representations are sustained in time by reverberating connections between pyramidal neurons (Romo et al., 1996; Merchant et al., 2003) through activation of NMDA receptors (Wang et al., 2013). To further explore the static coding of grip in M1, we developed a generalization index as a measure of the temporal generalization of grip encoding across preparatory and executive periods of the task by the same neural population (Crowe et al., 2010, 2014; Gámez et al., 2019). We found that grip encoding generalizes across both periods of the task despite the temporal differences in movement timing between animals. Therefore, our results indicate that M1 neural populations can keep a sustained coding of grip type during preparation and execution of movement. We did not observe this during force coding, which was more dynamic. We expected dynamic force coding, since the cueing of the pulling force magnitude did not allow for explicit force preparation. In addition, the lack of temporal generalization in force decoding might be related to the limited modulation strength in M1 by the pulling force magnitude. Taken together, these results suggest that the encoding of task-relevant movement parameters in M1 can be embedded in distinct coding schemes that change flexibly, according to task demands. A previous study has shown that static and dynamic coding schemes can coexist within the same cortical area (i.e., the prefrontal cortex), and it is possible to switch from one to another according to the task requirements and learning (Ceccarelli et al., 2023).

In summary, we found that grip type and force level are encoded as independent parameters in M1 neural activity during grasping at both single-unit and population levels. Coding of grip type was carried by both mixed-selective and grip-selective neurons, while force was encoded by mixed-selective neurons mostly. These neural modulations do not seem to be associated with stereotyped muscle activation patterns of each movement. In both animals, grip type was more strongly encoded than force and such coding scheme was static, engaging similar neural populations across task epochs. On the other hand, force encoding was short lived. This indicates that M1, like some associative areas such as the prefrontal cortex (Merchant et al., 2011; Ceccarelli et al., 2023), can switch from a static to a dynamic coding scheme in response to task demands. Furthermore, we consider it important to remark that for this study, we used a public database, generously shared to the scientific community by the laboratory of Dr. Alexa Riehle. This promotes data analyses that employ a variety of metrics and advances our knowledge of the neural bases of grasping behavior in nonhuman primates. This practice is becoming more popular and will enormously potentiate systems neuroscience (Milham et al., 2020; Messinger et al., 2021; Hartig et al., 2023).

## Synthesis

Reviewing Editor: Mark Laubach, American University Decisions are customarily a result of the Reviewing Editor and the peer reviewers coming together and discussing their recommendations until a consensus is reached. When revisions are invited, a fact-based synthesis statement explaining their decision and outlining what is needed to prepare a revision will be listed below. The following reviewer(s) agreed to reveal their identity: Alexander Kraskov. Note: If this manuscript was transferred from JNeurosci and a decision was made to accept the manuscript without peer review, a brief statement to this effect will instead be what is listed below.

Thank you for sending your work to eNeuro. The paper was reviewed by one expert, who was provided with reviews from your submission to JN. They raised some issues with the paper that we hope you can address through some revisions. Their full comments are given below. Please note that their comments are with regards to the previous reviews and your responses to them. Please revise to address all points that were raised.

Reviewer comments

I was invited to review this manuscript after two previous reviewer's with authors response to these reviews available to me as well.

After reviewing a revised manuscript and authors responses to the two previous reviewer's comments, I find paper technically sound and mostly well written but I also agree with previous reviewers that the presented findings and interpretations are quite similar to the previously published studies (e.g. macaque monkey study by Intveld et al 2018 and human study by Rastogi et al., 2021). This concern were attempted to be addressed by authors highlighting the differences throughout the revised manuscript and in the response to the reviewers in 4 points. Unfortunately, I do not find the authors responses convincing. See below the detailed discussion.

Overall, I find this paper an important confirmation of Intveld et 2028 results, especially important because Intveld et al had only one animal M1 dataset (although more sessions were recorded). But there are still methodological issues to be addressed.

Looking specifically at these points of differences

1. Single unit analysis.

a. I am not sure whether ROC analysis combined with permutation test presented by the authors is much different from Intveld et al "Cluster-based permutation test". The output measures (auROC -0.5) vs cluster summed F-statistics are different but both give an idea about a single unit contribution to encoding of grasp and force parameters.

b. "the diminished representation of pulling force at the population level compared to grip, can be explained by M1 containing fewer force-coding neurons, as well as such neurons carrying less pulling force information and only for brief periods of time." This statement seems to be circular, smaller representation is due to smaller number of (representing) neurons and smaller information (which is also representation) in each neuron.

c. "while the coding of grip was achieved through both grip-selective and mixed-selective neurons for both parameters, pulling force coding was only performed by mixed-selective neurons."

It is not at all surprising that grip coding is achieved through grip selective and mix-selective (which are also grip selective) neurons. It is also not surprising that force coded by mixed selective neurons (because they are force selective as well). It seems that the statement, as it is written, implies that force selective neurons (pure ones, not mixed selective ones) are not encoding force (seems odd and therefore surprising) but was not tested because there were just 2 neurons in each monkey that show force selectivity.

If more neurons would have been recorded, one can be pretty certain that force selective neurons will be encoding force, therefore, the apparent difference between grip and force encoding implied by "while ... both, ... only ..." disappears.

d. "there was no dependent modulation of single cell encoding for grip type and pulling force, namely, the direction of selectivity of neurons was independent between the two parameters." This seems to be indeed novel, but I am not entirely sure whether it was correctly tested.

i. To get \theta different from 90 degrees (inclined ellipse, e.g. ~45 degrees) one need to have neurons with preference to LF and PG and neurons with preference to HF and SG, or for \theta ~135 degrees preference (low FR) for low force to go along with preference (low FR) for SG and neurons with preference to HF and PG. Essentially, authors showed that the above scenario is not the case. But if ones assumes no specific direction in preference and just looks at the strength of modulation (abs(auROC_grip) vs abs(auROC_force)) one might be able to see whether strong modulation (~strong encoding/representation) for grip comes along with strong modulation for force.

ii. Why 400ms and 100ms were chosen as a point for testing independence of encoding?

iii. Why the same time point was chosen for force and grip, it could be that encoding happening in the same neurons (not independent) but at different time points

e. "The lack of significant interaction between grip type and pulling force in the demixed PCA corroborated this finding at the neural population level."

Wasn't this shown in Intveld et al? See similarities and corresponding results for the interaction between figures 5DE (Moreno et al ) and 7E (Intveld)

2. Temporal stability of grip and pulling force representations.

a. "We assessed how stable in time were the neural representations of grip and pulling force. Using cross- temporal decoding analysis we found that the M1 population can maintain a long- lasting grip representation before and during movement execution. Conversely, the pulling force population signal was transient and occurred mainly during movement execution."

It is not clear to me whether this was assessed in the best possible way. Strength of representation seems to be a confounding factor. Stability implies similarity, small change, but what is analysed instead is how strong is cross-time decoding. There is not much of force encoding even on diagonal and therefore it quickly diminishes to chance level on off diagonal. Specifically, looking at the decoding matrices (6B, 7B, BTW, not sure why diagonal elements were set to 0 rather than to values from figure 6A and 7A) one can deduce that although grip decoding is higher overall (i.e. off diagonal elements) there is a lot of change from diagonal to off diagonal for grip decoding vs force decoding. In 7B one can even see values close to 0 (bluish)which presumably correspond to very consistent (and likely significant) errors in decoding side grip as precision grip and vice versa, meaning that representation is not stable at all it actually flips at some points.

b. "The transient encoding and decoding of force in M1 contrast with the tonic and long-lasting application of force through the grasping device by the monkeys." Not really novel, it is well known that hold period (static period) of any grasp usually shows smaller firing rate than reach to contact (dynamic period).

3. Not sure how this one is different from 2

4. Certainly novel, and should be encouraged. As a suggestion, authors of this manuscript should consider sharing their codes to reproduce the figures of this manuscript alongside the data.
