# Supplementary figures and images for: Time Varying Encoding of Grasping Type and Force in the Primate Motor Cortex
Source: eNeuro. 2025 Apr 25;12(4):ENEURO.0010-25.2025. doi: 10.1523/ENEURO.0010-25.2025 (PMC12037165; doi:10.1523/ENEURO.0010-25.2025)

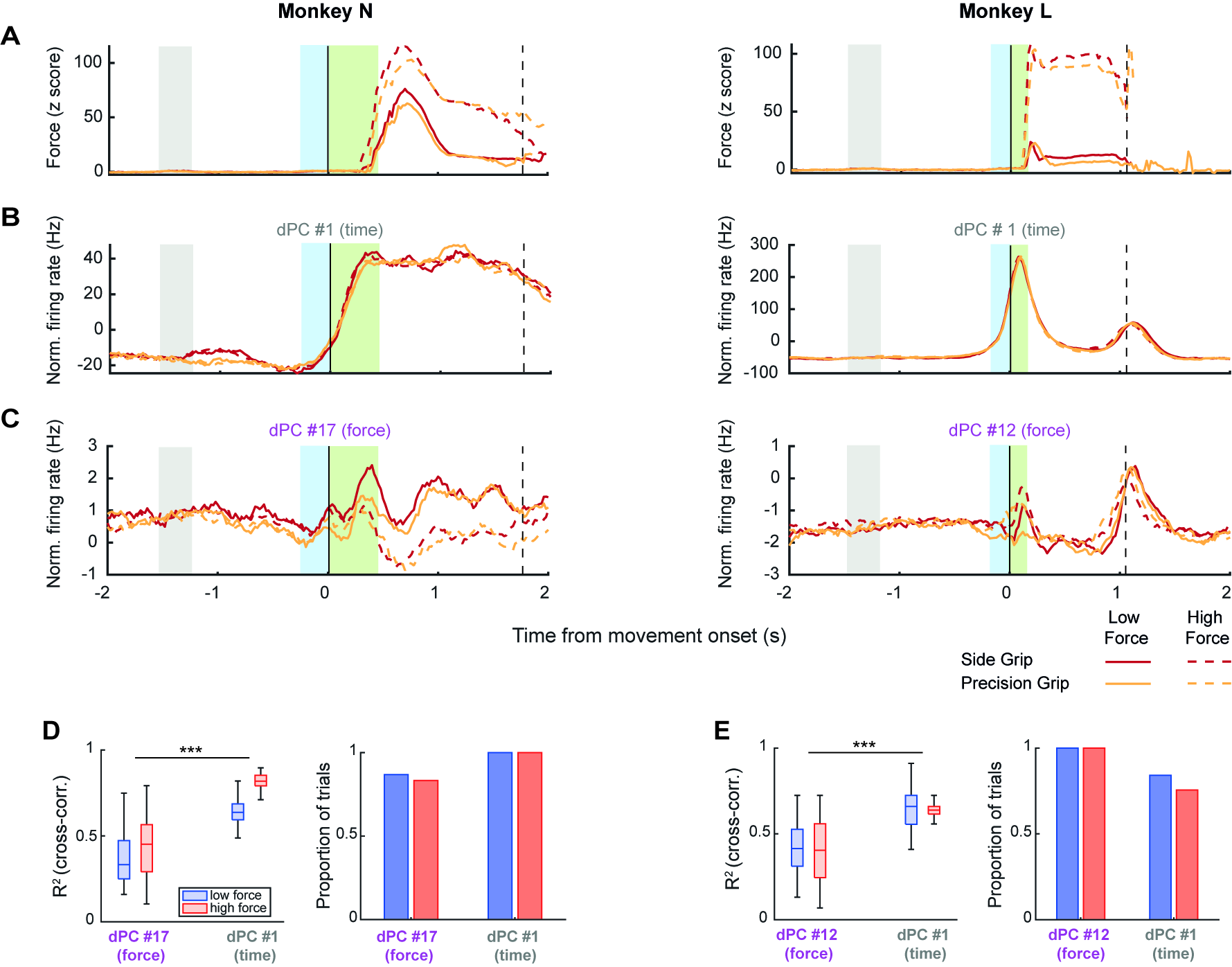

Supplement: Figure 5-1 — Correlation between the monkeys’ pulling force and condition-independent and force-related dPCs from M1 neurons. A: Normalized average pulling force signal, as a function of time for monkey N (left) and monkey L (right). B: Condition-independent (time) dPC that explained the most neural variance in monkey N and L, respectively. C: Force dPC that explained the most neural variance for monkey N (dPC #17) and monkey L (dPC #12). D (left): Absolute value of the maximum cross-correlation coefficient (R2) between each trial force signal and its related dPC (force and time), for low force (blue box) and high force (red box) trials, for monkey N. Black lines with asterisks indicate significant differences between marginalizations (2-way ANOVA, F(1,279) = 481.68, p = 0). D (right): Proportion of trials with a significant value (outside of the 99%-confidence bounds) of R2 when comparing the force signal between grip and force-related dPCs for monkey N. E: same as in D for monkey L (2-way ANOVA, F(1,327) = 233.35, p = 0). Download Figure 5-1, TIF file. [file eneuro-12-ENEURO.0010-25.2025-s001.tif]
